# Association between quality domains and health care spending across physician networks

**DOI:** 10.1371/journal.pone.0195222

**Published:** 2018-04-03

**Authors:** Farah Rahman, Jun Guan, Richard H. Glazier, Adalsteinn Brown, Arlene S. Bierman, Ruth Croxford, Therese A. Stukel

**Affiliations:** 1 Institute for Clinical Evaluative Sciences, Toronto, Ontario, Canada; 2 Li Ka Shing Knowledge Institute, St. Michael’s Hospital, Toronto, Ontario, Canada; 3 Department of Family and Community Medicine, Faculty of Medicine, University of Toronto, Toronto, Ontario, Canada; 4 Institute of Health Policy, Management and Evaluation, University of Toronto, Toronto, Ontario, Canada; 5 Dalla Lana School of Public Health, University of Toronto, Toronto, Ontario, Canada; 6 Center for Evidence and Practice Improvement, Agency for Healthcare Research and Quality, Rockville, Maryland, United States of America; 7 Lawrence S. Bloomberg Faculty of Nursing and Department of Medicine, Toronto, Ontario, Canada; 8 The Dartmouth Institute for Health Policy and Clinical Practice, Dartmouth College, Hanover, New Hampshire, United States of America; 9 Sunnybrook Research Institute, Sunnybrook Health Sciences Centre, Toronto, Ontario, Canada; Harvard Medical School, UNITED STATES

## Abstract

One of the more fundamental health policy questions is the relationship between health care quality and spending. A better understanding of these relationships is needed to inform health systems interventions aimed at increasing quality and efficiency of care. We measured 65 validated quality indicators (QI) across Ontario physician networks. QIs were aggregated into domains representing six dimensions of care: screening and prevention, evidence-based medications, hospital-community transitions (7-day post-discharge visit with a primary care physician; 30-day post-discharge visit with a primary care physician and specialist), potentially avoidable hospitalizations and emergency department (ED) visits, potentially avoidable readmissions and unplanned returns to the ED, and poor cancer end of life care. Each domain rate was computed as a weighted average of QI rates, weighting by network population at risk. We also measured overall and sector-specific per capita healthcare network spending. We evaluated the associations between domain rates, and between domain rates and spending using weighted correlations, weighting by network population at risk, using an ecological design. All indicators were measured using Ontario health administrative databases. Large variations were seen in timely hospital-community transitions and potentially avoidable hospitalizations. Networks with timely hospital-community transitions had lower rates of avoidable admissions and readmissions (r = -0.89, -0.58, respectively). Higher physician spending, especially outpatient primary care spending, was associated with lower rates of avoidable hospitalizations (r = -0.83) and higher rates of timely hospital-community transitions (r = 0.81) and moderately associated with lower readmission rates (r = -0.46). Investment in effective primary care services may help reduce burden on the acute care sector and associated expenditures.

## Introduction

Achieving high-value health care requires simultaneously improving population health, improving the individual’s experience of care, and reducing per capita costs of care.[1] The Triple Aim framework developed by the Institute for Healthcare Improvement recognizes that these components are interdependent, requiring a balanced focus on improving the quality and efficiency of services. Organizations often find it challenging to improve patient quality of care and health outcomes even with sufficient resources.[2] If we are to achieve a high-value health care system, we must understand how spending and quality are related in order to know where increased spending is likely to improve quality, but also where savings are possible without adversely affecting, and preferably improving, quality.

We used naturally-occurring Ontario multispecialty physician networks as our unit of performance measurement.[3] These virtual networks reflect groups of primary care and specialist physicians who are associated by virtue of sharing care for a common set of patients and admitting patients to the same hospital so that the networks mimic the populations served by Accountable Care Organizations (ACOs). They are small enough to detect meaningful variations in rates of processes and outcomes but large enough to have relatively stable rates over time. The characteristics of these networks, panel size, physician supply and assignment mechanism have been previously described.[3] With the passage of Ontario’s *Patients First Act* in 2016, responsibility for planning and performance improvement for the primary health care system will devolve to smaller regional levels to better address the unique health care needs of the province’s diverse urban, rural and remote communities. Much of the groundwork for this localized planning was undertaken by two of the authors using Ontario health administrative data and our physician network patient assignment mechanism.

In a *Chartbook*, we reported the performance of Ontario multispecialty physician networks on 65 quality indicators that reflect health care delivery in primary, specialty, acute, and long-term care, as well as timely transitions from the hospital or emergency department (ED) to the community.[4] The indicators chosen are amenable to intervention, measureable across the continuum of care from population health to primary care to tertiary care, and based on validated definitions derived from Ontario health administrative databases. While the *Chartbook* reported wide variability in quality indicators across physician networks, associations between quality and spending were not investigated.

The current study examines the association between health care quality and spending across physician networks within Ontario’s universal health care system. We also assessed associations between overall and outpatient primary care spending and potentially avoidable admissions, readmissions, and timely hospital-community transitions since previous work has shown associations between high rates of primary care supply and lower rates of mortality and hospitalizations for ambulatory care sensitive conditions.[5–9]

## Methods

### Physician networks

A total of 77 networks, serving 98.5% of the population, were included in the analyses. Two children’s hospital networks were excluded from non-paediatric indicators, the psychiatric hospital network was excluded from non-mental health indicators, and one remote network was excluded from all indicators due to extremely small population size. In this ecological study, the unit of analysis was the physician network since this is the natural functional and organizational locus of accountability for population-based care, as networks comprise large groups of physicians that share patients, and are therefore more conducive to system interventions and accountability than are individual physicians or practices.

### Quality indicators and quality domains

Quality Indicators (QIs) were based on events occurring during the two-year period between April 1, 2010 and March 31, 2012. Details on the definitions, data sources, diagnostic and procedure billing codes as well as the clinical guidelines used in the development of each indicator are reported in the *Chartbook*.[4] Timely transitions were measured as the percentage of patients with a follow-up visit to a primary care physician or relevant specialist within seven days of discharge, and shared care as follow-up visits with both a primary care physician and a relevant specialist within 30 days of discharge. Timely hospital-community transitions can result in fewer medical errors, improved communication between care providers, and improved health promoting behaviors at home.[10–13]

QIs were aggregated into six quality domains or clinical composites of screening and prevention, evidence-based medications, timely hospital-community transitions, potentially avoidable hospitalizations and emergency department (ED) visits, potentially avoidable readmissions and unplanned returns to the ED, and poor cancer end of life (EOL) care.[4] Domain rates for each physician network were calculated as the weighted average of the constituent indicator rates, weighting each by its denominator, the target population, as in other studies.[14] Rates of screening and poor cancer end-of-life care were not adjusted since they apply to the entire target population. Rates of hospitalization and readmissions were fully risk-adjusted using previously validated methods.[15–20] The remaining rates were indirectly standardized for age and sex.

### Health care spending

Costs of insured health care services were computed based on standardized provincial prices to reflect resources used.[21] Costs were those paid by Ontario’s Ministry of Health and Long-Term Care; patient out-of-pocket costs were not included. Mean per capita costs were calculated for each network over a two-year period (2010–2011), adjusted for age and sex, annualized, and expressed in 2011 Canadian dollars. Health care spending was computed for hospital, physician (overall and separately for primary care physicians and specialists), prescription (for those over age 65 years), and long-term care sector. Spending for outpatient primary care services was computed based on primary care physician claims for office visits, seeing patients in long-term care facilities, home visits and consultations through phone calls. In exploratory analyses, we decomposed primary care outpatient spending per capita into comprehensive primary care physician full time equivalents (FTEs) per capita (primary care supply) and outpatient primary care billings per primary care physician (primary care intensity).[22]

### Network characteristics

We explored network characteristics of rurality and marginalization to determine their association with healthcare quality. Network rurality was measured using the Rurality Index of Ontario (RIO), which accounts for population size and travel time, to categorize networks as urban (RIO 0–9), nonurban (RIO 10–39) and remote (RIO ≥ 40).[23] Population marginalization was measured using a census-based, empirically derived, theoretically-informed tool.[24] Briefly, marginalization is a process that creates inequalities along multiple axes of social differentiation. We report two dimensions, material deprivation (education, lone-parent families, receipt of government transfer payments, unemployment, low-income status, and dwellings in need of major repair) and dependency (proportion of the population aged 65 and older, dependency ratio, and proportion of population not participating in the labour force) to capture different aspects of marginalization. Both were calculated at the level of the census dissemination area, neighbourhoods with populations between 400 and 700 people.

### Data sources

Residents’ records were linked using unique, anonymized, encrypted identifiers across multiple Ontario health administrative databases containing information on all publicly insured, medically necessary hospital and physician services. Databases included the Discharge Abstract Database for hospital admissions, ICU admissions, procedures and transfers and includes the most responsible diagnosis for length of stay, secondary diagnosis codes, comorbidities present upon admission, complications occurring during the hospital stay, and attending physician identifier; the Ontario Mental Health Reporting System database for admissions to mental health–designated hospital beds; the National Ambulatory Care Reporting System for ED visits; the Ontario Health Insurance Plan (OHIP) for physician billings that includes diagnosis codes and procedures, and location of visit; the Ontario Drug Benefits for outpatient drug prescriptions for those over age 65 years; the Ontario Marginalization Index for multiple dimensions of marginalization in urban and rural Ontario; the Registered Persons Database for patient demographic information and deaths; and the Institute for Clinical Evaluative Sciences Physician Database which contains yearly information on all physicians in Ontario.

### Analysis

We report median and 10th and 90th percentile quality domain rates, weighted by target network populations. We considered a domain rate to have low variability if the ratio of the weighted 90th to 10th percentile across networks was less than 1.25, moderate variability if this ratio was between 1.25 and 2.0, and high variability if this ratio was greater than 2.0. The associations between quality of care and per capita population costs were evaluated using Pearson correlation coefficients, weighting by physician network denominators. For each domain, we computed the intraclass correlation coefficient (ICC) using multilevel logistic regression models, with response to the individual quality indicators as the dependent variable, adjusting for patient risk factors and individual quality indicators as fixed effects, and including random effects for physician networks to account for the clustered nature of the data since patients are nested within networks.[25] Since the domain ICCs were small, there was negligible attenuation of the correlations between domain rates.[25] All analyses were performed using SAS version 9.3. Research ethics approval was obtained from the institutional review board at Sunnybrook Health Sciences Centre, Toronto, Ontario, Canada.

## Results

Individual quality indicators, domain rates and their variations across physician networks are reported in Table 1. The quality indicator rates and their variations across physician networks were discussed extensively in the *Chartbook*, so we provide a brief overview only.[4] Rates of prescribing of evidence-based medications were very good with little variation across networks. Rates of receipt of recommended screening and preventive care were good except for HbA1c testing for those with diabetes. Timely hospital-community transitions demonstrated moderate to high variability across physician networks. About half of patients discharged from hospital with a cardiac condition or pediatric asthma, and one-third of those with a psychiatric or non-cardiac chronic condition were seen by a physician within seven days. Rates of shared care were low. The highest rates of readmission and return to ED after discharge were observed for congestive heart failure (CHF) patients. Most cancer patients received home care visits in the last 6 months of life, but moderate to high variability was seen in their receipt of chemotherapy, ED visits and intensive care unit stays.

**Table 1:** Quality indicator rates, according to quality domain.

Quality Indicator	Risk Adjustment	Median^‡^	10th to 90th Percentiles^‡^	Ratio of 90th to 10th Percentiles	Interclass Correlation Coefficient (ICC)
**Screening and prevention, %**		64.9	60.4–68.1	1.13	0.007
Eye examination for individuals with diabetes	Unadjusted	69.5	66.1–74.7	1.13	0.014
Cholesterol testing for individuals with diabetes	Unadjusted	87.9	84.2–90.0	1.07	0.028
HbA1c testing for individuals with diabetes	Unadjusted	41.7	36.1–50.6	1.40	0.020
Optimal screening (eye examination, cholesterol test, HbA1c test) for individuals with diabetes	Unadjusted	34.1	30.0–42.5	1.42	0.018
Bone mineral density test, eligible females	Unadjusted	83.9	74.2–90.3	1.22	0.083
Bone mineral density test after a fracture, males	Age-sex	11.7	6.0–16.9	2.82	0.036
Bone mineral density test after a fracture, females	Age-sex	20.4	12.9–25.8	2.00	0.029
Mammogram, eligible females	Unadjusted	66.9	62.4–71.1	1.14	0.007
Pap test, eligible females	Unadjusted	72.1	68.4–77.0	1.13	0.012
Colorectal cancer screening, eligible individuals	Unadjusted	61.2	55.8–67.4	1.21	0.015
Post-stroke therapy provided as a part of home care	Age-sex	65.0	43.4–79.1	1.82	0.086
**Evidence-based medications, %**		75.0	72.7–77.8	1.07	0.003
ACE or ARB after AMI hospitalization	Age-sex	79.4	72.9–84.5	1.16	0.014
Beta-blocker after AMI hospitalization	Age-sex	79.5	71.8–84.3	1.17	0.022
Statin after AMI hospitalization	Age-sex	89.4	84.9–93.9	1.11	0.026
ACE or ARB after CHF hospitalization	Age-sex	69.8	61.9–74.9	1.21	0.009
Beta-blocker after CHF hospitalization	Age-sex	69.5	61.5–76.1	1.24	0.018
Statin after CHF hospitalization	Age-sex	63.7	55.9–69.4	1.24	0.013
Antihypertensive after stroke hospitalization	Age-sex	84.9	77.2–90.3	1.17	0.023
Statin after stroke hospitalization	Age-sex	76.7	70.0–84.7	1.21	0.017
ACE or ARB for individuals with diabetes	Age-sex	72.0	69.9–75.3	1.08	0.004
Antihypertensive for individuals with diabetes	Age-sex	84.5	82.4–86.8	1.05	0.006
Statin for individuals with diabetes	Age-sex	69.6	65.9–72.4	1.10	0.006
**Timely Hospital to community transitions, %**		43.5	31.1–51.7	1.66	0.051
Office visit* within 7 days after discharge for AMI	Age-sex	45.5	35.4–54.7	1.55	0.025
Office visit* within 7 days after discharge for CHF	Age-sex	46.4	33.3–53.9	1.62	0.033
Office visit* within 7 days after discharge for psychiatric care	Age-sex	32.0	19.2–39.6	2.06	0.035
Office visit* within 7 days after discharge for COPD, diabetes, asthma, pneumonia or unstable angina	Age-sex	35.8	26.9–46.7	1.74	0.029
Office visit,* newborn, within 7 days after discharge	Unadjusted	80.2	55.7–87.1	1.56	0.167
Office visit,* pediatric, within 7 days after discharge for asthma	Age-sex	46.4	24.3–59.3	2.44	0.085
Office visit,* pediatric, within 7 days after high-triage-level ED visit for asthma	Age-sex	24.3	13.5–31.3	2.32	0.058
Shared care,^†^ pediatric, within 30 days after discharge for asthma	Age-sex	8.5	3.8–18.7	4.92	0.060
Shared care,^†^ pediatric, within 30 days after high-triage-level ED visit for asthma	Age-sex	3.9	1.9–5.6	2.95	0.032
Shared care^†^ within 30 days after discharge for AMI	Age-sex	24.2	13.9–35.8	2.58	0.070
Shared care^†^ within 30 days after discharge for CHF	Age-sex	27.1	12.9–36.4	2.82	0.092
Shared care^†^ within 30 days after discharge for psychiatric care	Age-sex	19.2	9.1–24.1	2.65	0.087
Office visit* within 7 days after high-triage-level ED visit for atrial fibrillation, angina, CHF or asthma	Age-sex	39.7	28.9–48.3	1.67	0.035
**Adverse outcomes: potentially avoidable admissions and ED visits**		11.6	8.3–17.6	2.12	0.018
Hospitalization for acute complication of diabetes, %^§^	Fully risk-adjusted	0.5	0.3–0.7	2.33	0.026
Hospitalization for chronic complication of diabetes, %^§^	Fully risk-adjusted	3.9	3.3–4.7	1.42	0.008
Hospitalization for asthma, per 1,000 individuals with asthma^§^	Fully risk-adjusted	1.3	0.9–2.0	2.22	0.000
Hospitalization for diabetes, per 1,000 individuals with diabetes^§^	Fully risk-adjusted	5.1	3.4–7.2	2.12	0.031
Hospitalization for CHF, per 1,000 individuals with CHF^§^	Fully risk-adjusted	48.8	40.0–64.5	1.61	0.014
Hospitalization for COPD, per 1,000 individuals with COPD^§^	Fully risk-adjusted	70.1	52.8–90.4	1.71	0.024
ED visit for acute complication of diabetes, per 1,000 individuals with diabetes^§^	Fully risk-adjusted	29.1	19.7–46.2	2.35	0.052
ED visit for chronic complication of diabetes, per 1,000 individuals with diabetes^§^	Fully risk-adjusted	12.5	10.1–17.2	1.70	0.015
**Adverse outcomes: 30-day readmissions and ED visits, %**		17.9	16.7–19.7	1.18	0.012
Readmission within 30 days after discharge for AMI	Fully risk-adjusted	12.2	9.0–14.2	1.58	0.008
Readmission within 30 days after discharge for CHF	Fully risk-adjusted	20.0	16.6–24.3	1.46	0.004
Readmission within 30 days after discharge for stroke	Fully risk-adjusted	9.6	6.9–11.2	1.62	0.003
Readmission within 30 days after discharge for psychiatric care	Fully risk-adjusted	13.6	11.7–17.6	1.50	0.023
ED visit within 30 days after discharge for AMI	Fully risk-adjusted	23.0	20.3–29.2	1.44	0.011
ED visit within 30 days after discharge for CHF	Fully risk-adjusted	29.5	25.7–36.6	1.42	0.008
ED visit within 30 days after discharge for stroke	Fully risk-adjusted	17.1	13.5–20.8	1.54	0.004
ED visit within 30 days after discharge for psychiatric care	Fully risk-adjusted	22.8	19.7–27.4	1.39	0.039
**Poor cancer end-of-life care, %**		30.6	27.0–35.6	1.32	0.016
Died in hospital (excluding recipients of palliative care)	Unadjusted	36.9	24.5–52.6	2.15	0.075
No home care visit in last 6 months of life	Unadjusted	21.3	16.2–27.6	1.70	0.022
No palliative care in last 6 months of life	Unadjusted	38.1	26.0–56.5	2.17	0.077
ICU stay in last 2 weeks of life	Unadjusted	7.3	5.4–9.5	1.76	0.005
ED visit in last 2 weeks of life	Unadjusted	33.9	29.6–41.8	1.41	0.013
Chemotherapy in last 2 weeks of life	Unadjusted	3.0	1.5–4.6	3.07	0.022
No house call in last 2 weeks of life	Unadjusted	78.2	67.1–84.9	1.27	0.061
**Spending**					
Total spending per capita, $	Age-sex	2,540	2,257–2,868	1.27	
Hospital spending per capita, $	Age-sex	986	824–1,234	1.50	
Total physician spending per capita, $	Age-sex	543	476–613	1.29	
Primary care physician spending per capita, $	Age-sex	203	158–239	1.51	
Specialist spending per capita, $	Age-sex	347	289–397	1.37	
Prescription drug spending per capita, age 65+, $	Age-sex	320	262–369	1.41	
Long-term care spending per capita, $	Age-sex	250	187–286	1.53	

*Office visit: at least one office visit with a primary care provider or appropriate specialist. Includes visits by a physician to a patient’s home or long-term care facility, and telephone calls to a patient.

^†^Shared care: at least one office visit with both a primary care provider and appropriate specialist. Includes visits by a physician to a patient’s home or long-term care facility, and telephone calls to a patient.

^§^Based on an annualized rate.

^‡^Quality indicator values were weighted by the network denominators.

ACE: angiotensin converting enzyme inhibitor; ARB: angiotensin receptor blocker; AMI: acute myocardial infarction; CHF: congestive heart failure; COPD: chronic obstructive pulmonary disease; ED: emergency department; ICU: intensive care unit; ODB: Ontario Drug Benefit.

**Fig 1:**
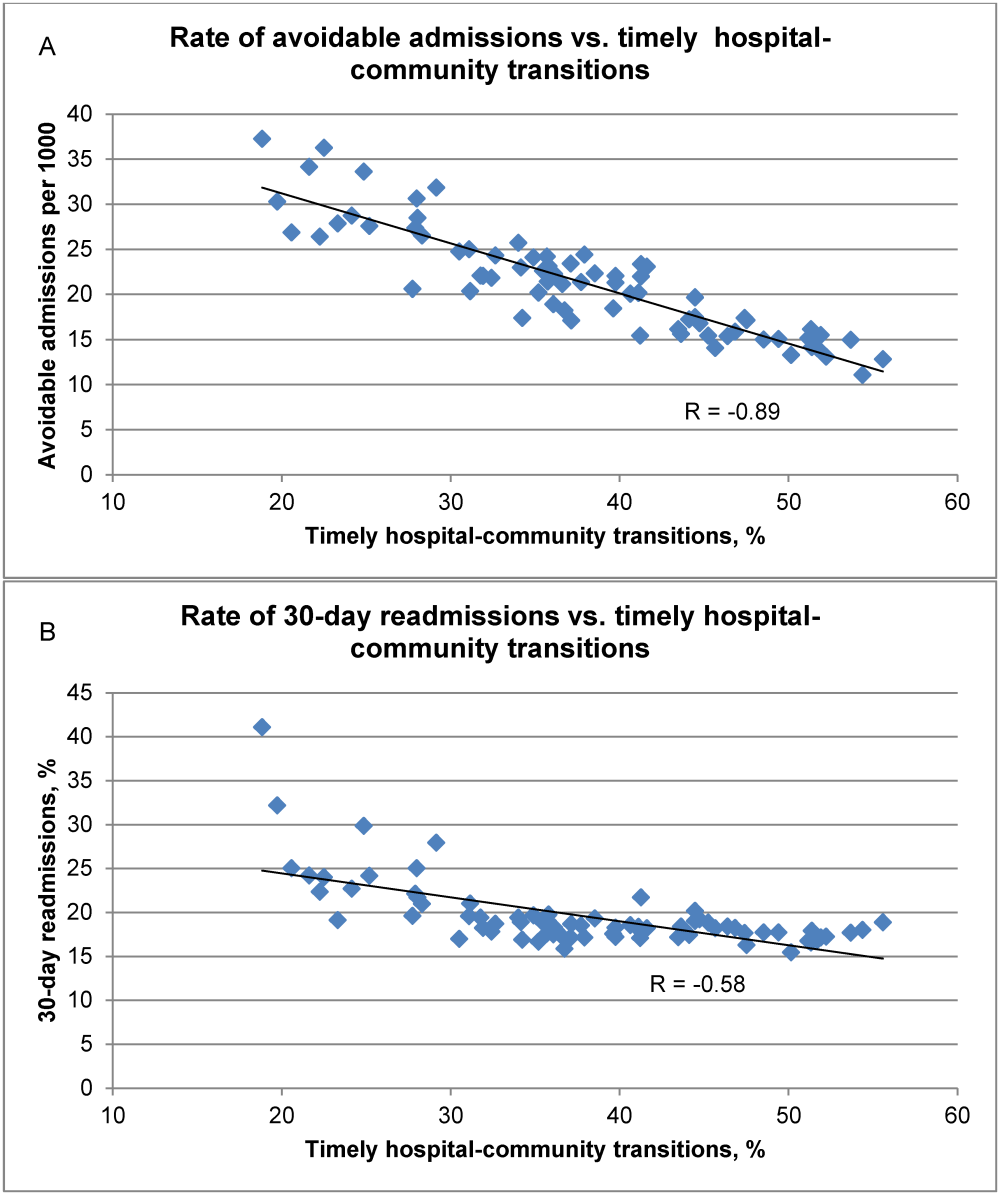
Rates of avoidable admissions per 1000 patients and 30-day readmissions (%) vs. timely hospital-community transitions (%). Rates of avoidable admissions and 30-day readmissions are fully risk-adjusted across all quality indicators within these domains. Quality indicators comprising timely hospital-community transitions were all age-sex adjusted, except for office visit for a newborn within 7 days after hospital discharge.

Correlation coefficients between the quality domain rates are reported in Table 2 and the relationships displayed in Fig 1. Many relationships were as expected such as strong associations between rates of avoidable admissions, readmissions and poor EOL care. However, we also found that rates of timely hospital-community transitions were inversely associated with rates of admissions (r = -0.89), readmissions (r = -0.58) and poor EOL care (r = -0.52) (Table 2, Fig 1).

**Table 2:** Correlations between quality domain rates.

	**Screening and prevention**	**Evidence-based medications**	**Timely hospital-community transitions**	**Potentially avoidable admissions**	**30-day readmissions**	**Poor end-of-life care**
**Screening and prevention**	1.0	-0.06	0.29	-0.31	-0.36	-0.38
		p = 0.62	p = 0.01	p = 0.007	p = 0.002	p<0.001
**Evidence-based medications**		1.0	-0.09	0.11	0.02	0.07
			p = 0.46	p = 0.37	p = 0.87	p = 0.53
**Timely Hospital-community transitions**			1.0	**-0.89**	**-0.58**	**-0.52**
				**p<0.001**	**p<0.001**	**p<0.001**
**Potentially avoidable admissions**				1.0	**0.66**	**0.54**
					**p<0.001**	**p<0.001**
**30-day readmissions**					1.0	**0.47**
						**p < 0.001**
**Poor end-of-life-care**						1.0

**Fig 2:**
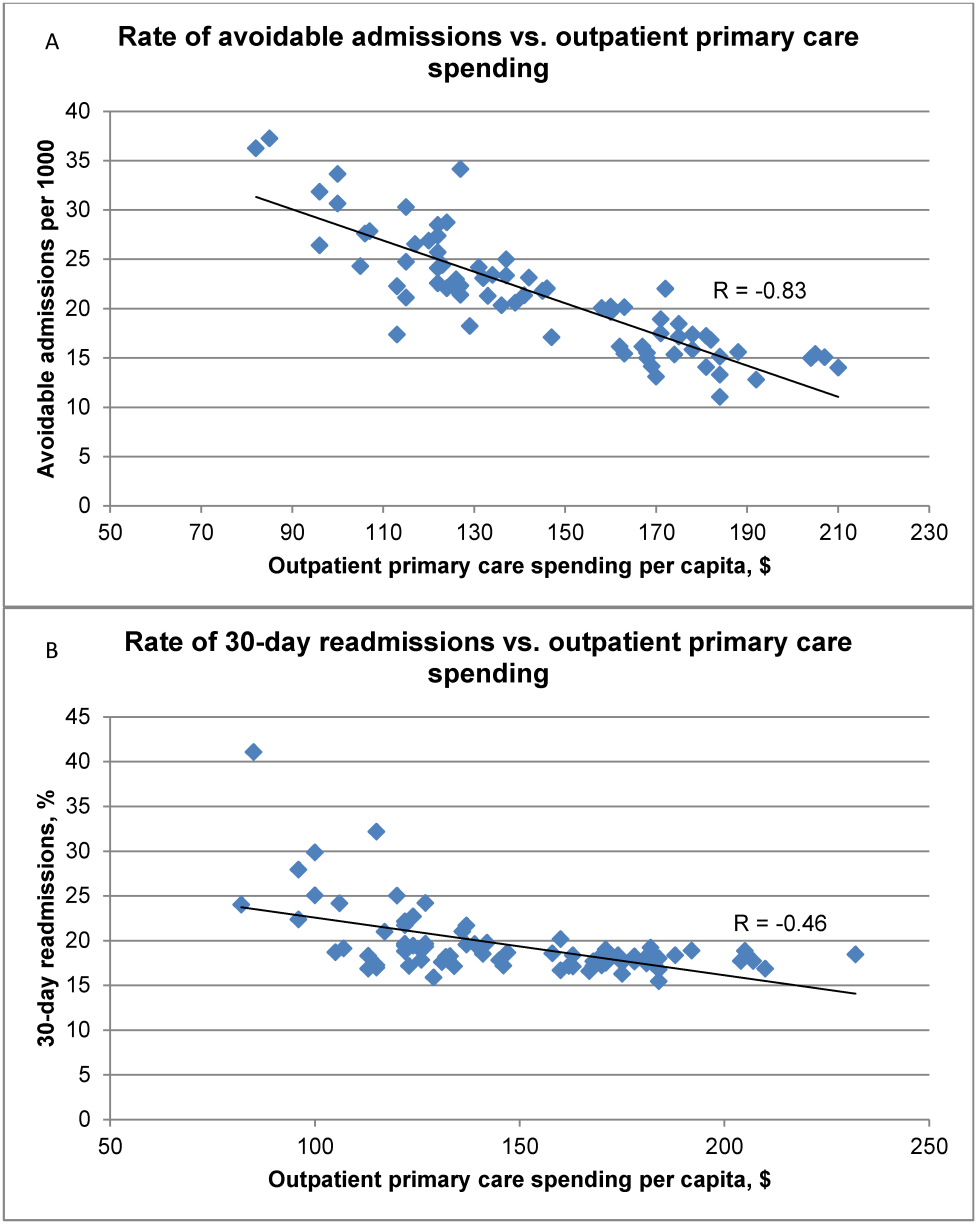
Rates of avoidable admissions per 1000 patients and 30-day readmissions (%) vs. outpatient primary care spending per capita. Rates of avoidable admissions and 30-day readmissions are fully risk-adjusted across all quality indicators within this domain. Outpatient primary care spending was age-sex adjusted.

**Fig 3:**
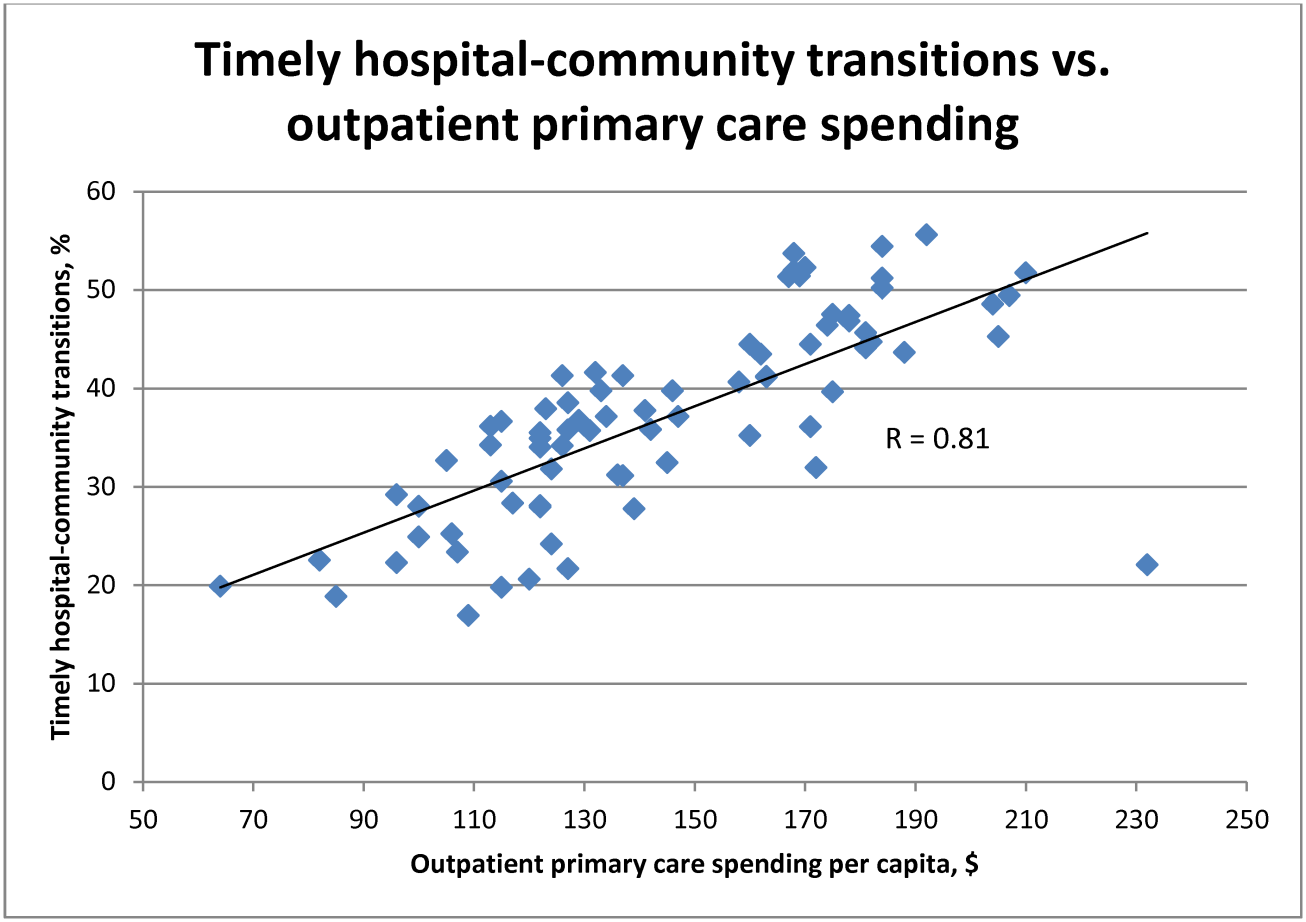
Timely hospital-community transitions (%) vs. outpatient primary care spending per capita. Quality indicators comprising timely hospital-community transitions were age-sex adjusted, except for office visit for a newborn within 7 days after hospital discharge. Outpatient primary care spending per capita was age-sex adjusted.

Networks with higher rates of physician spending had lower rates of avoidable admissions; the strongest association, however, was with outpatient primary care physician spending (r = -0.83) (Table 3, Fig 2). Networks with higher outpatient primary care spending also had lower readmission rates (r = -0.46) and more timely hospital-community transitions (r = 0.81) (Table 3, Figs 2 and 3). Networks with higher rates of timely hospital-community transitions had lower spending on prescription drugs and long-term care (r = -0.56 and -0.61, respectively). As expected, there were strong relationships between spending and hospital admission rates; however, we found little association between spending and rates of prescribing of evidence-based medications, and screening and prevention.

**Table 3:** Correlations between spending and quality domain rates.

	**Screening and prevention**	**Evidence-based medications**	**Timely hospital-community transitions**	**Risk-adjusted potentially avoidable admissions**	**Risk-adjusted 30-day readmissions**	**Poor end-of-life care**
**Hospital spending**	-0.39	0.12	**-0.78**	**0.84**	0.64	0.50
	p<0.001	p = 0.32	**p<0.001**	**p<0.001**	p<0.001	p<0.001
**Physician spending**	-0.01	0.01	**0.63**	**-0.60**	-0.27	-0.32
	p = 0.93	p = 0.92	**p<0.001**	**p<0.001**	p = 0.02	p = 0.005
**Primary care physician spending**	-0.14	0.03	**0.52**	**-0.48**	-0.05	-0.18
	p = 0.25	p = 0.79	**p<0.001**	**p<0.001**	p = 0.65	p = 0.13
**Primary care outpatient spending**	-0.007	-0.06	**0.81**	**-0.83**	**-0.46**	-0.38
	p = 0.95	p = 0.63	**p<0.001**	**p<0.001**	**p<0.001**	p = 0.001
**Specialist spending**	0.07	-0.005	**0.49**	**-0.49**	-0.31	-0.31
	p = 0.57	p = 0.97	**P<0.001**	**p<0.001**	p = 0.007	p = 0.007
**Prescription drug spending**	-0.35	0.32	**-0.56**	**0.50**	0.34	0.45
	p = 0.002	p = 0.005	**p<0.001**	**p < 0.001**	p = 0.003	p<0.001
**Long-term care spending**	-0.30	0.23	**-0.61**	**0.61**	0.35	0.48
	p = 0.008	p = 0.05	**p<0.001**	**p<0.001**	p = 0.002	p<0.001

In exploratory analyses, we found that primary care ambulatory spending per capita was more highly related to primary care intensity (r = 0.77) than to primary care supply(r = 0.20). Furthermore, primary care intensity was also associated with lower admission and readmission rates (r = -0.64 and r = -0.32, respectively) and higher rates of timely transitions (r = 0.64), whereas overall primary care supply was unrelated to these domains.

Rurality, dependency and material deprivation were associated with higher rates of potentially avoidable admissions and readmissions, and inversely associated with timely hospital-community transitions, as expected. Greater dependency and material deprivation were also associated with poor EOL care (Table 4).

**Table 4:** Correlations between network characteristics and quality domain rates.

	**Screening and prevention**	**Evidence-based medications**	**Timely hospital-community transitions**	**Risk-adjusted potentially avoidable admissions**	**Risk-adjusted 30-day readmissions**	**Poor end-of-life care**
**Non-urban network**	-0.19	0.02	**-0.58**	**0.70**	**0.65**	**0.39**
	p = 0.11	p = 0.89	**p<0.001**	**p<0.001**	**p<0.001**	**p<0.001**
**Dependency**	-0.21	0.13	**-0.72**	**0.77**	**0.57**	**0.56**
	p = 0.07	p = 0.27	**p<0.001**	**p<0.001**	**p<0.001**	**p<0.001**
**Material deprivation**	-0.61	0.18	**-0.46**	**0.43**	**0.51**	**0.49**
	p<0.001	p = 0.12	**p<0.001**	**p<0.001**	**p<0.001**	**p<0.001**

## Discussion

We found that physician networks with higher rates of timely hospital-community transitions had lower rates of potentially avoidable readmissions, and avoidable admissions. We found that outpatient primary care spending was strongly associated with higher rates of timely hospital-community transitions and lower rates of avoidable admissions, and moderately associated with lower readmissions rates. In addition, timely transitions were related to lower spending on pharmaceuticals and long-term care.

The costs and savings associated with quality improvements are multifaceted and complex in nature, and are spread out across stakeholders and across time. Systems need to ensure that healthcare providers have the incentives and support to implement quality improvement initiatives that span sectors.[2] Policies that encourage a link between cost and quality in only one sector, like primary care or hospitals, are unlikely to be successful in realizing those savings, which has stimulated the need for cross-sectoral integrated networks. High quality, lower cost care has been achieved by large U.S. multispecialty physician group practices through the redesign of care to meet the needs of chronic disease patients by strengthening primary care, implementing chronic disease management programs, and integration of care.[26–29]

The U.S. is experimenting with promising initiatives in integrated delivery systems such as Accountable Care Organizations (ACOs), groups of providers that are accountable for the quality of care of a defined population and collectively share in the savings of more efficient delivery of services.[30] There is evidence that such reforms may contribute to increasing quality while slowing spending growth, although there are many challenges to achieving these objectives.[31–34] While these formal associations are uncommon in Canada, health care providers form informal networks, such as those in our study, based on sharing patients and, often, information.[3,35] The finding that primary care outpatient spending was associated with lower preventable hospital care is consistent with recent findings from ACOs showing better cost performance with primary care-run ACOs.[36]

Best practices recommend seeing a primary care physician shortly after discharge to allow for monitoring and evaluating patients’ progress during this vulnerable and high-risk period.[37] Care coordination in the primary care setting has been identified as a key strategy to improve the effectiveness, efficiency and safety of the health care system, and includes improved transitions of care, communicating and knowledge sharing, monitoring and follow-up, and assessing patients’ needs and goals.[38] Improving transitions through pre-discharge interventions (patient education, discharge planning), post-discharge interventions (timely follow-up), and provider continuity may reduce 30-day readmissions.[37,39] Other work has found that hospitalizations for ambulatory care sensitive conditions might be prevented if outpatient care were provided in an effective and timely manner in an ambulatory care setting.[40,41]

This study suggests that the effect of primary care supply on outcomes may be driven more by primary care intensity than primary care supply (headcounts), thereby extending the findings of Starfield et al. and underscoring the need to identify what aspects of primary care practice lead to better outcomes.[5–8] Current indicators are crude measures of primary care performance, and others have suggested that these traditional quality improvement indices may not be useful for identifying changes in quality or variations in outcomes.[14,42] As more meaningful measures are developed, there will be a need to assess the relationship of the new measures to the outcomes we examined. In addition, one-size-fits-all measures may not be appropriate for all patients and there is a need to align measures with patient goals and preferences. For example, tight diabetes control in a frail elder may increase the risk of adverse outcomes, and some cancer screening measures may not be appropriate in those with limited life expectancy. This resonates with many primary care physicians who are suspect of linear disease-specific targets when their patients are highly complex and often make idiosyncratic choices. Investing in primary care may require increased time spent conversing with patients, especially those with multimorbidity, which may not improve technical quality but can motivate patients to make better decisions about their health, adhere to treatment plans, increase use of outpatient interdisciplinary team care, and increase communication among physicians.[43–45] Additionally, our findings suggest that increased investment in primary care may be needed to optimize individual and population health outcomes.[46]

Prior research on the relationship between health care quality and overall spending has produced inconsistent results.[47–53] The Commonwealth Fund reported widespread variability across US Hospital Referral Regions (HRRs) and suggested that better access to care was associated with higher quality of care and better patient outcomes.[54,55] A systematic review that appraised the evidence for an association between health care costs and quality among 61 US-based studies reported that associations were inconsistent, and that the impact of spending on quality was small to moderate.[56] It concluded that future studies should focus on which types of spending are most effective in improving quality. A large US longitudinal cohort study showed large, persistent variations in health care quality and spending across HRRs but found that higher spending regions had neither better quality of care nor increased survival.[57,58] In contrast, a similarly designed longitudinal cohort study in Canada showed that higher spending Ontario hospitals had lower mortality and readmission rates, and higher quality of care.[59]

Our study has a number of strengths. The study is population based and is unique in its breadth of indicators evaluated and their associations with sector-specific spending. We investigated the association between quality and spending across Ontario physician networks, which reflect populations of patients that share physicians similarly to US Accountable Care Organizations (ACOs) and are, therefore, a potential locus of accountability for chronic disease care.

Several limitations should be considered. The study design is ecological so that causal relationships cannot be inferred. This study was meant to reveal patterns and not to demonstrate causality; such associations would need to be confirmed in longitudinal cohort studies using the individual patient as the unit of analysis. This study may be generalizable to the Canadian universal health care system, but these relationships may differ in other countries’ health care systems. As in all observational studies, residual confounding due to unmeasured patient risk factors could have influenced the results. We also could not investigate patient experience of care.

Reducing spending without decreasing quality involves targeting poor hospital-community coordination, wasteful spending, and ineffective care through programs that provide incentives for value-based care provision, such as bundled payments and integrated health care systems, which encourage coordination and integration, and more aggressively targeting preventable hospitalizations by bolstering primary and ambulatory care.[60] Preventing hospital admissions and readmissions, improving continuity of care and managing health care spending are complex issues requiring multi-faceted care and action from all levels of the health care system. Strengthening outpatient primary care and developing integrated models of primary care that extend beyond the medical home to the medical neighborhood with linkages between population health and community services are key elements to optimizing patient health and reducing health care costs. Future research should focus on studying the effects of timely transitions on reducing adverse events using longitudinal cohort studies.
